# Valproic Acid Induces Antimicrobial Compound Production in *Doratomyces microspores*

**DOI:** 10.3389/fmicb.2016.00510

**Published:** 2016-04-13

**Authors:** Christoph Zutz, Markus Bacher, Alexandra Parich, Bernhard Kluger, Agnieszka Gacek-Matthews, Rainer Schuhmacher, Martin Wagner, Kathrin Rychli, Joseph Strauss

**Affiliations:** ^1^Institute for Milk Hygiene, University of Veterinary Medicine ViennaVienna, Austria; ^2^Research Platform Bioactive Microbial Metabolites, Bioresources and Technologies Campus in TullnTulln an der Donau, Austria; ^3^Division of Chemistry of Renewables, Department of Chemistry, University of Natural Resources and Life Sciences, ViennaTulln an der Donau, Austria; ^4^Center for Analytical Chemistry, Department of Agrobiotechnology (IFA-Tulln), University of Natural Resources and Life Sciences, ViennaTulln an der Donau, Austria; ^5^Fungal Genetics and Genomics Unit, Department of Applied Genetics and Cell Biology, University of Natural Resources and Life Sciences, ViennaTulln an der Donau, Austria; ^6^Health and Environment Department, Bioresources, Austrian Institute of Technology GmbH, University and Research Campus TullnTulln an der Donau, Austria

**Keywords:** fungi, *Doratomyces*, valproic acid, diketopiperazine, antimicrobial compounds

## Abstract

One of the biggest challenges in public health is the rising number of antibiotic resistant pathogens and the lack of novel antibiotics. In recent years there is a rising focus on fungi as sources of antimicrobial compounds due to their ability to produce a large variety of bioactive compounds and the observation that virtually every fungus may still contain yet unknown so called “cryptic,” often silenced, compounds. These putative metabolites could include novel bioactive compounds. Considerable effort is spent on methods to induce production of these “cryptic” metabolites. One approach is the use of small molecule effectors, potentially influencing chromatin landscape in fungi. We observed that the supernatant of the fungus *Doratomyces* (*D.*) *microsporus* treated with valproic acid (VPA) displayed antimicrobial activity against *Staphylococcus* (*S*.) *aureus* and two methicillin resistant clinical *S. aureus* isolates. VPA treatment resulted in enhanced production of seven antimicrobial compounds: cyclo-(L-proline-L-methionine) (cPM), *p*-hydroxybenzaldehyde, cyclo-(phenylalanine-proline) (cFP), indole-3-carboxylic acid, phenylacetic acid (PAA) and indole-3-acetic acid. The production of the antimicrobial compound phenyllactic acid was exclusively detectable after VPA treatment. Furthermore three compounds, cPM, cFP, and PAA, were able to boost the antimicrobial activity of other antimicrobial compounds. cPM, for the first time isolated from fungi, and to a lesser extent PAA, are even able to decrease the minimal inhibitory concentration of ampicillin in MRSA strains. In conclusion we could show in this study that VPA treatment is a potent tool for induction of “cryptic” antimicrobial compound production in fungi, and that the induced compounds are not exclusively linked to the secondary metabolism. Furthermore this is the first discovery of the rare diketopiperazine cPM in fungi. Additionally we could demonstrate that cPM and PAA boost antibiotic activity against antibiotic resistant strains, suggesting a possible application in combinatorial antibiotic treatment against resistant pathogens.

## Introduction

One of the biggest challenges in healthcare today is the rising number of antibiotic resistant pathogenic microorganisms isolated from human and veterinary sources and the lack of novel antimicrobial agents in the discovery pipeline (Garcia-Alvarez et al., 2012). This has reignited the interest in the search of antimicrobial compounds derived from natural sources (Ling et al., 2015). Fungi have gained great attention as source of antimicrobial agents due to their ability to produce a large variety of bioactive compounds and the observation that virtually every fungus may still contain yet unknown so called “cryptic,” often silenced, compounds (Keller et al., 2005). The fungus *Doratomyces* (*D*.) *microsporus* is a member of the Microascales family and is closely related to *Scopulariopsis* spp., which is known to produce the potent antimicrobial agent deacetoxycephalosporin C (Webber et al., 1969). *D. microsporus* is a worldwide distributed fungus mainly isolated from feces but also from rotting plant material and soil, which has been associated with decay (Domsch et al., 2007). It is known to produce an extracellular keratinase, which is closely related to proteinase K (Vignardet et al., 1999; Gradisar et al., 2000). Furthermore it has been shown that this fungus has the ability to degrade the antifungal alkaloid sampangine (Orabi et al., 1999). Besides these studies little is known about the primary and secondary metabolite profile of this fungus.

In fungi the expression and production of natural products, including antimicrobial products, is either part of the primary metabolism and/or the secondary metabolism. The primary metabolism comprises compounds which are essential for the growth of the fungus and the secondary metabolism includes compounds not essential for the survival of the fungus, but holding a competitive advantage in the respective habitat. In fungi the secondary metabolite related genes are often physically linked, forming so-called gene clusters, and expression is strongly influenced by biotic and abiotic environmental factors (Shwab et al., 2007). Thus “cryptic” compounds may not have been identified in previous antimicrobial activity screens due to the complex pattern of biotic and abiotic factors necessary to induce the production of these compounds. Under standard laboratory conditions the secondary metabolism related gene clusters are often transcriptionally silenced, which has been linked to the formation of facultative heterochromatin. The switch between transcriptional silenced heterochromatin and active euchromatin is accomplished by reversible chemical modifications of histones (Wu and Grunstein, 2000; Hayes and Hansen, 2001). Activation of transcription is mainly linked to acetylation of histones which is performed by histone acetyltransferases (HATs) while deacetylation, carried out by histone deacetylases (HDACs), is known to generally decrease secondary metabolite levels by the formation of heterochromatin (Csordas, 1990; Brownell and Allis, 1996; Strauss and Reyes-Dominguez, 2011; Gacek and Strauss, 2012). Recently, the ability of small molecule effectors, like valproic acid (VPA), to inhibit the catalytic activity of HDACs and to modulate the subsequent “cryptic” expression profile of secondary metabolites has been shown in fungi (Shwab et al., 2007; Cole, 2008; Cichewicz, 2010; Hagemann et al., 2011). Furthermore our previous study suggests that the effect of VPA seems to be target specific in fungi (Zutz et al., 2013).

In a previous work we have established a screening system based on the use of such low molecular weight molecules, like VPA, to induce the production of “cryptic” antimicrobial compounds in fungi (Zutz et al., 2014). In this work we focused on one hit candidate, the fungus *D*. *microsporus*, which displayed antimicrobial activity exclusively after VPA treatment. In the current study we identified and characterized the “cryptic” antimicrobial active compounds of the fungus *D. microsporus* after induction by VPA treatment. We report the identification of seven induced antimicrobial compounds derived from primary and secondary metabolism of the fungus, including cyclo-(L-proline-L-methionine), which was for the first time discovered in fungi. Thus the results indicate that VPA is a potent tool to induce antimicrobial compound production.

## Materials and Methods

### Strains and Chemicals

The *D. microsporus* isolate used in this study originated from soil and was obtained from the fungal strain collection of the AIT (Austrian Institute of Technology, Fungal Genetics and Genomics Unit). Identification of the fungus was performed according to Klaubauf et al. (2010). Bacterial strains used in this study are listed in **Table 1**. The extended-spectrum beta-lactamase (ESBL) *Klebsiella* (*K*.) *pneumoniae* (B100173) isolate is resistant to the β-lactam antibiotics Ampicillin, Amoxicillin and Piperacillin, the cephalosporins Cefotaxim and Ceftazidim, the aminoglycoside antibiotics Gentamicin and Tobramycin, the monobactam antibiotic Aztreonam, the tetracyclines, sulfamethoxazole-trimethoprim, the fluoroquinolones and Chloramphenicol. The ESBL *Escherichia* (*E*.) *coli* (B300129) isolate is resistant to the β-lactam antibiotics Ampicillin, Amoxicillin, and Piperacillin, the cephalosporins Cefotaxim and Ceftazidim, the aminoglycoside antibiotic Tobramycin, the monobactam antibiotic Aztreonam, the flouroquinolones, the tetracyclines and sulfamethoxazole-trimethoprim. The methicillin-resistant *Staphylococcus* (*S.*) *aureus* isolate (MRSA) B337919 is resistant to β-lactam antibiotics, the macrolides Azithromycin and Erythromycin and the isolate B335466 was resistant to β-lactam antibiotics and the fluoroquinolones Enrofloxacin, Marbofloxacin and Ciprofloxacin (all resistant strains were kindly provided by Analyze Biolab GmbH, Austria). VPA was obtained from Sigma Aldrich (Germany) and prepared as recently described (Zutz et al., 2013). 5-acetamidopentanoic acid was obtained from Vitas-M laboratory (USA), the diketopiperazines cyclo-(L-proline-L-methionine) and cyclo-(L-phenylalanine-L-proline) were obtained from APARA Bioscience (Germany). The chemicals *p*-hydroxybenzaldehyde, phenyllactic acid, indole-3-carboxylic acid and indole-3-acetic acid were obtained from Sigma–Aldrich (Germany) and phenylacetic acid (PAA) from Merck (Germany).

**Table 1:** Strains used in this study.

Species	Strain	Source
*Staphylococcus* (*S.*) *aureus*	ATCC 6538	Human
*Pseudomonas* (*P.*) *aeruginosa*	ATCC 9027	Human
*Candida* (*C.*) *albicans*	ATCC 10231	Human
*Klebsiella* (*K.*) *pneumoniae*	ATCC 13883	Human
*Escherichia* (*E.*) *coli*	Roche3943B (MC 1061)	Human
*Staphylococcus* (*S*.) *epidermidis*	ATCC 12228	Human (skin)
*Enterococcus* (*E*.) *faecium*	DSMZ 13589	Human (faces)
*Enterococcus* (*E*.) *faecalis*	ATCC 29212	Human (urine)
*Streptococcus* (*S*.) *pneumoniae*	DSMZ 11865	Human
*Clostridium* (*C*.) *difficile*	DSMZ 1296	Human (feaces)
*Listeria* (*L*.) *monocytogenes*	ATCC BAA-679 (EGDe)	Rabbit
*Streptococcus* (*S*.) *suis*	HK47	Human
*Bacillus* (*B*.) *cereus* (isolate)	HK48	Food
ESBL *Klebsiella* (*K*.) *pneumoniae*	B100173	Human
ESBL *Escherichia* (*E*.) *coli*	B300129	Human
MRSA *Staphylococcus* (*S.*) *aureus*	B337919	Human
MRSA *Staphylococcus* (*S.*) *aureus*	B335466	Human

### Preparation of Fungal Extract

The fungal strain was inoculated on malt extract agar (MEA; Oxoid, USA) and incubated at room temperature. Spores were harvested after 10 days of incubation and inoculated in 20 ml minimal fungal media at a concentration of 10^6^ spores/ml (Moser media, composed of 10 g/l glucose, 0.2 g/l yeast extract, 2 g/l tryptic-digested peptone from casein, 0.5 g/l KH_2_PO_4_, 50 mg/l inositol, 75 mg/l CaCl_2_, 10 mg/l FeCl_3_, 150 mg/l MgSO_4_, 10 mg/l MnSO_4_). Tryptophan (Trp) was added at a concentration of 2 × 10^3^ μM. VPA was added at a final concentration of 50 μM. The fungal culture was incubated for 72 h at room temperature on a rotary shaker at 180 rpm. After incubation the supernatant was filtered through a cellulose filter and was extracted twice with ethylacetate 1:1 (v/v). The ethylacetate phases were pooled and dried under reduced pressure and stored at -20°C. For isolation of compounds in total 100 liters of fungal culture were prepared in 50 2-l cultures under identical conditions and extracted.

### Assessment of Antimicrobial Activity

The antimicrobial activity testing was performed as previously described (Zutz et al., 2014). Additionally to the indicator organisms *S. aureus, Candida* (*C.*) *albicans* and *Pseudomonas* (*P.*) *aeruginosa* we used also *Klebsiella* (*K.*) *pneumoniae, Staphylococcus* (*S*.) *epidermidis, Enterococcus* (*E*.) *faecium, Enterococcus* (*E*.) *faecalis, Streptococcus* (*S*.) *pneumoniae, Streptococcus* (*S*.) *suis, Clostridium* (*C*.) *difficile, Listeria* (*L*.) *monocytogenes, Bacillus* (*B*.) *cereus, E. coli*, ESBL *Klebsiella* (*K*.) *pneumoniae*, ESBL *Escherichia* (*E*.) *coli* and two MRSA *Staphylococcus* (*S.*) *aureus* (MRSA 335466, MRSA 337919). As control we used the indicator organisms without the fungal culture.

Stability of antimicrobial activity was assessed after proteinase K digestion and heat treatment. Proteinase K digestion was performed by incubating the fungal extract either with 1 × 10^5^ μg/l proteinase K at 37°C for 1 week or incubating the extract with 1 × 10^6^ μg/l proteinase K at 37°C for 24 h. Heat stability was assessed after incubation of the fungal extract at 95°C for 24 h.

### Cell Viability Assay

The effect of the fungal extract on the viability of the indicator organisms *S. aureus* and *P. aeruginosa* was determined using a cell staining assay (LIVE/DEAD BacLight Invitrogen). Samples were taken after 0, 6, and 18 h of incubation. Each sample was diluted with Fresenius Ringer 1000-fold. The filters were assembled according to manufacturer’s protocol (Swinnex Millipore). Each assembly consisted of a cellulose acetate membrane filter and a polycarbonate membrane filter (Sterlitech). The filters were equilibrated with 1 ml of Fresenius Ringer solution for 30 min at room temperature prior to use. The fluorescence probes propidium iodide and syto 9 were prepared according to manufacturer’s protocol. After mixing the probes with the indicator organisms, the mixture was incubated for 20 min in the absence of light at room temperature. Following the incubation step the suspension was diluted with Fresenius Ringer solution in a ratio of 1:0.8 and pressed through the filters. Examination was performed using a confocal laser scanning microscope (510 Meta, ZEISS; syto 9 excitation at 488 nm and emission at 505–530 longpass filter and propidium iodide excitation at 543 nm and emission at 610–640 longpass filter). For Syto9 the following parameters were used: pinhole 2 μm, laser gain 771–800, digital offset at -0.07, laser intensity at 5% and digital gain at 1. For propidium iodide the parameters were pinhole 2 μm, laser gain 540–600, digital offset at -0.07, laser intensity at 10% and digital gain at 1. Image analysis was performed using ImageJ (Schneider et al., 2012) and the Zen software (Zeiss). As control we used the indicator strains without addition of the fungal culture.

### Cytotoxicity Assay

Cytotoxicity of the fungal extract and the identified compounds was determined measuring lactate dehydrogenase release using human intestinal epithelial (Caco2) and human hepatocytic (HepG2) cells. Briefly, cells were cultivated using Eagle’s minimum essential media (MEM) supplemented with 2 mM L-glutamine, 10% fetal bovine serum (FBS), 1 × 10^5^ Units/l penicillin, 1 × 10^8^ μg/l streptomycin sulfate, 2.5 × 10^6^ μg/l amphotericin B and 1% non-essential amino acids (NEAA; all from PAA) at 37°C in a humidified atmosphere (95% relative humidity) containing 5% CO_2_. Cells were seeded into 96-well plates (5 × 10^4^ cells per well) and incubated until a confluent cell layer had developed. After discarding of the media cells were incubated with 50 μl of media and 50 μl of fungal extract or compound (final concentration: PAA 2 × 10^6^ μg/l, 4FP 2 × 10^6^ μg/l, ICA 2 × 10^6^ μg/l, IAA 2 × 10^5^ μg/l, PLA 2 × 10^6^ μg/l, cPM 2 × 10^6^μg/l and cFP 2 × 10^6^ μg/l) for 24 h at 37°C. Media alone and VPA (50 μM) were taken as control. LDH release was measured according to the manufacturer’s protocol (Sigma–Aldrich, USA). The percentage of dead cells was calculated using a standard curve of serial diluted lysed cells (100% dead cells) as previously described (Zutz et al., 2014).

In parallel the % of metabolic cells was measured using the XTT [2,3-Bis-(2-methoxy-4-nitro-5-sulfophenyl)-2H-tetra zolium-5-carboxanilide] assay (Thermo Scientific). Briefly, after treatment with the fungal extract or the different compounds cells were incubated with XTT and phenanzine methosulfate (Thermo Scientific) for 2 h. Absorbance was measured at 450 nm and % of metabolic active cells was calculated versus the control sample (media). As controls we incubated the cells without fungal cultures or compound, and with VPA alone.

### High Performance Liquid Chromatography (HPLC)

The dried fungal extract was re-dissolved in methanol/water (1:4) and fractionated on a preparative HPLC system (Agilent 1100 series) coupled with an ELSD (Sedex, Sedere 85 LT-ELSD, 3.1 bar nitrogen, 50°C) using a Gemini NX, 5 μm C18, 110Å, 150 × 21.2 mm AXIA (Phenomenex, USA) column. Separation was performed using a linear methanol/water gradient starting at 20–40% methanol in 20 min followed by a cleaning time of 4 min with 100% methanol at a flow rate of 20 ml min^-1^ with an injection volume of 1800 μl. Six pooled fractions of 53 individual separation runs, in total 630 ml per fraction, were collected, evaporated and dissolved in methanol/water (1:1). The antimicrobial active fractions were further separated on the preparative HPLC system using a Gemini NX, 5 μm C18, 110Å, 150 × 21.2 mm AXIA column. An isocratic separation was performed using acetonitrile/water (20:80) with 0.1% formic acid for 10 min at a flow rate of 20 ml min^-1^. Absorbance of fractions was measured at 210, 225, 250, and 300 nm. Bioactive fractions were stored at -20°C until further structure elucidation.

### Structure Elucidation

Structure elucidation of the isolated compounds was performed using nuclear magnetic resonance (NMR, Supplementary Table S3) and mass spectrometry (MS). NMR spectra were recorded on a Bruker Avance II 400 (Bruker, Rheinstetten, Germany; resonance frequencies 400.13 MHz for ^1^H and 100.61 MHz for ^13^C) equipped with a 5 mm observe broadband probe head (BBFO) with z–gradients at room temperature with standard Bruker pulse programs. The samples were dissolved in 0.6 ml of methanol-d_4_ (euriso-top, 99.8% D). The MS measurements were performed on an Orbitrap LTQ XL-MS (Thermo Scientific) with HPLC (Thermo Scientific) and HTC-Pal autosampler (PAL SYSTEM). Internal standards of purchased compounds were used to confirm the presence and the amount of the compounds in the fungal cultures.

### Determination of MIC

Minimal inhibitory concentrations (MICs) of purified compounds and commercially available standards were assessed against *S. aureus, C. albicans, P. aeruginosa, K. pneumoniae, E. coli*, ESBL *K*. *pneumoniae*, ESBL *E*. *coli* and the two MRSA strains (B337919 and B335466). Stock solutions of 5 × 10^6^ μg/l of *p*-hydroxybenzaldehyde, and phenyllactic acid were prepared using water. Stock solutions of 1 × 10^8^ μg/l indole-3-carboxylic acid, PAA and indole-3-acetic acid were prepared using methanol and diluted with water (working solution of 5 × 10^6^ μg/l). Stocks of 1 × 10^6^ μg/l of cFP and cPM were dissolved in water. Concentrations of 5 × 10^6^, 2.5 × 10^6^, 2 × 10^6^, 1.5 × 10^6^, 1 × 10^6^, 5 × 10^5^, 2.5 × 10^5^, 2 × 10^5^, 1 × 10^5^, 5 × 10^4^, 1 × 10^4^ μg/l were used for MIC determination. Methanol and water were used as control.

### Analysis of Histone Posttranslational Modifications by Western Blot

Mycelia from the *D. microsporus* cultures were harvested after 24, 48, and 72 h by filtration and frozen in liquid nitrogen. Histones were extracted under acidic conditions as described by Honda and Selker (2008). Samples were suspended in Laemmli’s SDS sample buffer and quantified with Pierce BCA Protein Assay (Thermo Scientific). Fifteen microgram of purified histones were separated on 15% SDS-PAGE gel and subsequently transferred to nitrocellulose membrane (GE Healthcare) by electroblotting. Histone H3 acetylation (H3Ac) was detected with primary antibody specific to H3Ac (Millipore, 06-599). As a loading control histone H3 C-terminus was detected with anti H3 C-terminus antibody (Abcam, 1791). The primary antibodies were identified with anti- rabbit (Sigma–Aldrich, A0545) horseradish peroxidase (HRP) conjugated secondary antibody. Chemiluminescence was detected with Clarity^TM^ ECL Western Substrate and ChemiDoc^TM^ XRS (Bio- Rad).

## Results

### Antimicrobial Activity of *D. microsporus*

**FIGURE 1:**
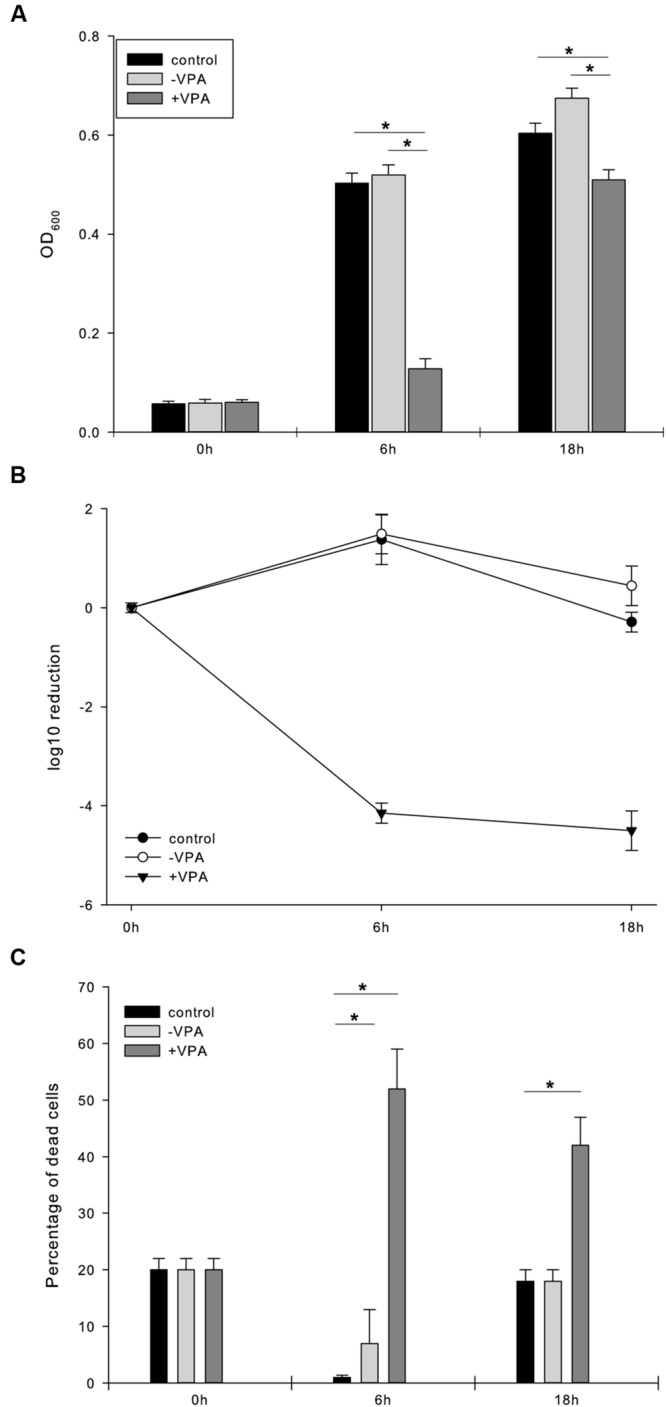
**Antimicrobial effect of the supernatant of valproic acid treated *D. microsporus* on *S. aureus*.** Effect of the supernatant of untreated (-VPA) and VPA treated (+VPA) *D. microsporus* on growth [OD_600_, **(A)**] and log10 CFU reduction **(B)** and percentage of dead cells **(C)** of *S. aureus* measured after 0, 6, and 18 h of incubation at 37°C. Control comprises of *S. aureus* cells grown without fungal extract. Data are presented as mean values ± standard deviations of three biological replicates performed in triplicate. Asterisks indicate statistically significant difference compared to the control (*p* < 0.05) according to student’s *t*-test.

The supernatants of the VPA treated *D. microsporus* cultures showed a strong antimicrobial activity against *S. aureus* (**Figure 1A**) and the MRSA strains B337919 and B335466 whereas the untreated fungal extracts displayed no antimicrobial activity. The active fungal extracts showed a log_10_ growth reduction of 3.7–4 log units for *S. aureus* after 6 h of incubation (**Figure 1B**). Furthermore a log reduction of 3.3–3.6 log units against both MRSA strains was observed. Weak activity was observed against *E. coli*, ESBL *E. coli* and *K. pneumoniae* (**Table 2**). The cell viability assay (LIVE/DEAD) revealed that the VPA treated fungal extracts led to 30% of dead cells after 6 h of incubation which indicated a bacteriostatic activity of the fungal culture (**Figure 1C**). To further characterize the antibacterial compound(s) in the extracts we determined proteinase K stability. The antimicrobial activity after incubation with 1 × 10^5^ μg/l for 24 h and with 1 × 10^6^ μg/l proteinase K at 37°C for 1 week (data not shown) (Supplementary Figure S1) was unchanged. This indicated that the active compound(s) were not proteins or peptides susceptible to proteinase K digestion. Heat stability of the fungal extracts was assessed after incubation at 96°C for 1 h. No significant reduction of bioactivity was observed (data not shown).

**Table 2:** Antimicrobial activity of fungal extracts.

	Growth inhibition^a^	
Strain	6 h	18 h
*S.aureus*	+++	++
*MRSA 337*	++	++
*MRSA 335*	++	++
*P. aeruginosa*	-	-
*C. albicans*	-	-
*E. coli*	+	-
*ESBL E. coli*	+	–
*K. pneumoniae*	+	+
*ESBL K. pneumoniae*	-	-
*L. monocytogenes*	-	-
*E. faecalis*	-	-
*E. faecium*	-	-
*B. cereus*	-	-
*S. epidermidis*	-	-
*S. suis*	+	-
*S. pneumoniae*	-	-

*^a^Growth inhibition was graded as follows: (+++) no measurable growth, (++) more than half growth reduction compared to control, (+) less than half reduction compared to control, (-) no significant reduction compared to control. Control comprises growth of test organism in the presence of supernatant of untreated fungus.*

### Cytotoxicity of *D. microsporus* Extract

**FIGURE 2:**
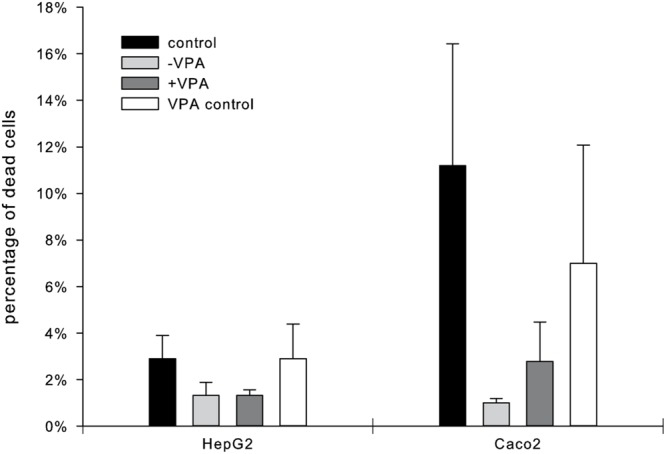
**Cytotoxic effect of the supernatant of VPA treated (+VPA) and untreated (-VPA) *D. microsporus* on human intestinal epithelial Caco2 and human hepatocytic HepG2 cells.** Control comprises HepG2 and Caco2 cells incubated in media for 24 h, VPA control comprises cells incubated with media containing VPA. Data, presented as percentage of dead cells, are mean values ± standard deviations of three biological replicates performed in triplicate.

Observed cytotoxicity using the LDH assay was below 1% of dead cells for Caco2 and HepG2 cells after 24 h which is regarded as non-cytotoxic for the mixture of unknown compounds (**Figure 2**). Overall we detected a lower cytotoxicity of cells incubated with the fungal cultures compared to the media, indicating that the fungal extracts contain compounds supporting the survival of human cells. VPA alone did not increase cytotoxicity compared to the media alone. The percentage of metabolic active cells measured with the XTT assay showed corresponding high rates (Supplementary Figure S2).

### Purification and Characterization of Compounds

**FIGURE 3:**
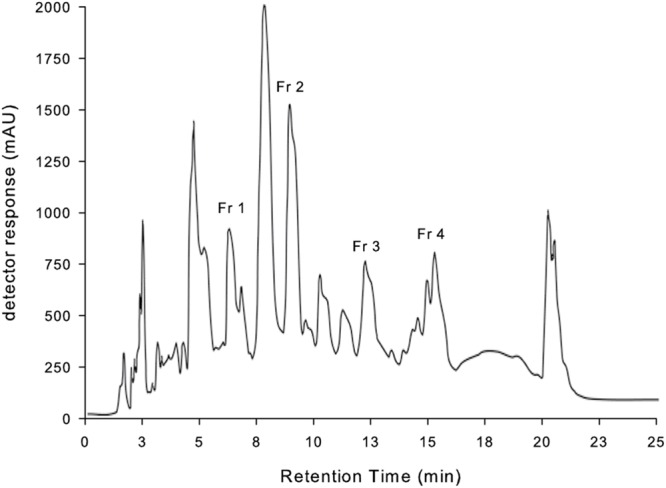
**High performance liquid chromatography (HPLC)-chromatogram of the EtOAc extract of the supernatant of VPA treated *D. microspores*.** Fraction 1 – 4 are antimicrobial active fractions.

**FIGURE 4:**
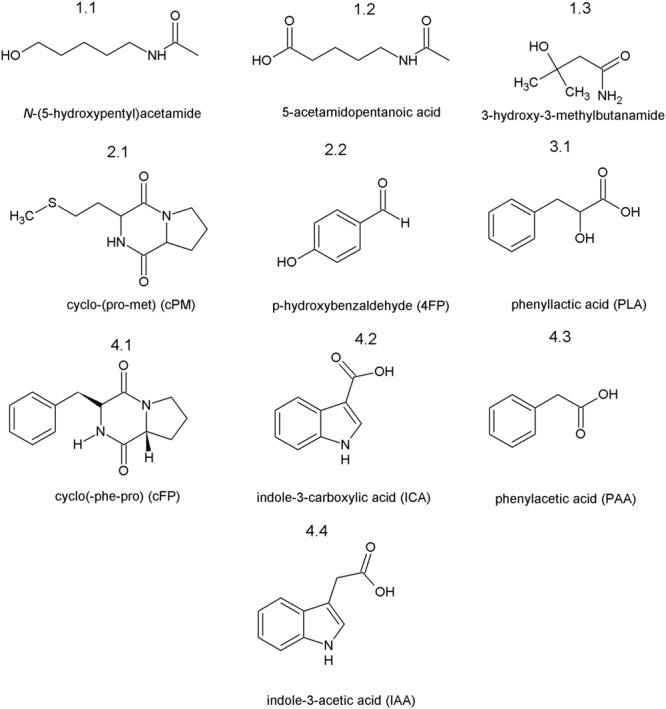
**Compounds isolated from the supernatant of the VPA treated *D. microsporus* culture.** Numbers indicate the corresponding fraction and peak of the isolated compounds.

Bioactivity driven fractionation revealed that the antimicrobial activity was recovered in the ethylacetate phase of the VPA treated fungal culture after liquid extraction. Further separation of the ethylacetate phase on a preparative reversed phase HPLC system using a C18 column and methanol as solvent resulted in the identification of four antimicrobial active fractions termed Fraction 1–4 (**Figure 3**). All fractions displaying antimicrobial activity were further separated using the C18 column and acetonitrile supplemented with 0.1% formic acid. Three compounds of fraction 1 could be identified: *N*-(5-hydroxypentyl)acetamide (1.1), 5-acetamidopentanoic acid (1.2) and 3-hydroxy-3-methylbutanamide (1.3); two compounds of fraction 2: diketopiperazine cyclo-(L-proline-L-methionine) (cPM, 2.1) and *p*-hydroxybenzaldehyde (4FP, 2.2); one compound of fraction 3: phenyllactic acid (PLA; 3.1) and four compounds of fraction 4: the diketopiperazine cyclo-(L-phenylalanine-L-proline) (cFP, 4.1), indole-3-carboxylic acid (ICA, 4.2), PAA (4.3) and indole-3-acetic acid (IAA, 4.4). Compound identification was based on NMR structure elucidation and confirmed by MS using internal standards (Supplementary Table S1). Additionally we confirmed the presence of the identified compounds in the crude fungal culture. **Figure 4** shows the structure formulas of the 10 isolated compounds.

### Determination of MICs

In total seven compounds showed an inhibitory effect on microbial growth. Generally, accepted MIC values for antibiotics in clinical use range from 0.003 (for levofloxacin) to 100 mg/l (for co-trimoxazole). However, in research MIC values for antibiotics are tested up to a concentration of 1 g/l (Rodloff et al., 2008).

Thus the threshold for considering a compound as being antimicrobial was set to MIC < 5 g/l. *S. aureus* showed susceptibility to IAA (MIC: 400 mg/l), which was also displayed by the MRSA strain B335466. However, the MRSA strain B337919 was more susceptible showing a MIC of 200 mg/l. IAA displayed also strong antimicrobial activity against *C. albicans* (MIC: 400 mg/l) (**Table 3**). The isolated diketopiperazines showed inhibitory activity on the growth of all tested organisms. Since MIC values were higher than 5 g/l we determined the lowest concentrations at which the inhibitory effect was detectable (**Table 4**).

**Table 3:** Minimal inhibitory concentration (MIC) values of isolated compounds.

Strain	MIC [g/l]				
	PAA	4FP	IAA	PLA	ICA
*S. aureus*	2	2	0.4	2	2
MRSA 337	2	2.5	0.2	2	2
MRSA 335	2	2.5	0.4	2	2
*P. aeruginosa*	n.d.	2.5	2	5	2
*C. albicans*	2.5	1	0.4	n.d.	n.d.
*E. coli*	2	2.5	1.75	2.5	1
ESBL *E. coli*	2	2.5	1.75	5	1
*K. pneumoniae*	2.5	2.5	0.4	2.5	2
ESBL *K. pneumoniae*	2.5	2.5	2	5	2

*n.d, not determinable.*

**Table 4:** Inhibitory concentrations of isolated diketopiperazines.

Strain	Inhibitory concentration [g/l]	
	cFP	cPM
*S. aureus*	2	2.5
MRSA 337	2	2.5
MRSA 335	2	2.5
*P. aeruginosa*	5	5
*C. albicans*	2.5	5
*E. coli*	5	5
ESBL *E. coli*	5	5
*K. pneumoniae*	3	5
ESBL *K. pneumoniae*	3	5

### Influence of VPA

As VPA is thought to boost production of secondary metabolites through inhibition of histone deacetylases, we determined acetylation levels of histone H3 K9 and K14 of VPA-treated and control cultures. However, we could not detect any significant increase in acetylation of the N-terminal lysines of histone 3 compared to the untreated control culture (Supplementary Figure S3). This suggests that either VPA does not increase histone acetylation in *D. microsporus* or that the chromatin effect is locus-specific and can thus not be detected by the applied method detecting only global changes in acetylation levels.

The influence of VPA on the production of the isolated compounds was determined by MS using commercially available standard compounds. It could be shown that VPA induces the production of all isolated compounds. PLA was not detectable in the untreated fungal culture, but VPA treatment resulted in the production of 0.07 mg/l PLA.

Two of the isolated compounds (IAA and ICA) contain indole structures and could have genetic links to the fungal auxin biosynthesis pathway in which Trp is used as main substrate (Maor et al., 2004). Therefore we determined the influence of Trp alone and in combination with VPA on the production of the seven antimicrobial compounds (**Table 5**). Trp addition to media showed increased production of all indole related compounds. The production of IAA, the final product of the biosynthesis pathway, was increased up to 43-fold. The combination of Trp and VPA lead to a further increase of the IAA production (57-fold) compared to the untreated culture. The production of both diketopiperazines and PLA were not influenced by Trp. In addition, the concentration of PAA decreased compared to VPA treated culture if Trp was used during cultivation indicating that Trp may be an inhibitor of the PAA biosynthetic pathway.

**Table 5:** Concentrations of compounds isolated from the supernatant of untreated, VPA treated (+VPA), tryptophan treated (+Trp) and VPA and tryptophan (VPA+Trp) treated *D. microspores.*

Compound	Amount [mg/l]				x-fold of untreated extract		
	Untreated	+VPA	+Trp	VPA + Trp	+VPA	+Trp	VPA + Trp
PAA	0.08	0.60	0.16	0.39	7.5	2.38	4.875
4FP	0.04	0.05	0.04	0.04	1.25	1	1
IAA	0.01	0.08	0.43	0.57	8	43	57
PLA	n.d.	0.07	n.d.	n.d.	n.d.	n.d.	n.d.
cPM	0.02	0.07	0.02	0.04	3.5	1	2
ICA	0.01	0.05	0.17	0.17	5	17	17
cFP	0.0008	0.0040	0.0007	0.0011	5	0.875	1.375
5-acetamido pentanoic acid	n.d.	0.02	n.d.	n.d.	n.d.	n.d.	n.d.
*N*-(5 hydroxy pentyl)acetamide	n.d.	0.01	n.d.	n.d.	n.d.	n.d.	n.d.
3-hydroxy-3-methylbutanamide	n.d.	0.004	n.d.	n.d.	n.d.	n.d.	n.d.

*n.d., not detectable.*

### Cytotoxicity of Isolated Compounds

Cytotoxicity of all purified compounds was determined using the determined MIC concentrations. PAA, ICA, cPM and cFP showed low toxicity (3.99–8.61% dead cells). Moderate cytotoxicity was displayed by 4FP against Caco2 cells (25.9% dead cells); in parallel the rate of metabolic active cells decreased compared to the control (57.3% metabolic active cells). PLA showed moderate cytotoxicity against the Caco2 cells (19.7% dead cells) and elevated cytotoxicity against HepG2 cells (54.63% dead cells). Accordingly, the rate of metabolic active cells ranged for both cell lines from 50 to 70% (Supplementary Table S2).

### Combinatorial Effects

**FIGURE 5:**
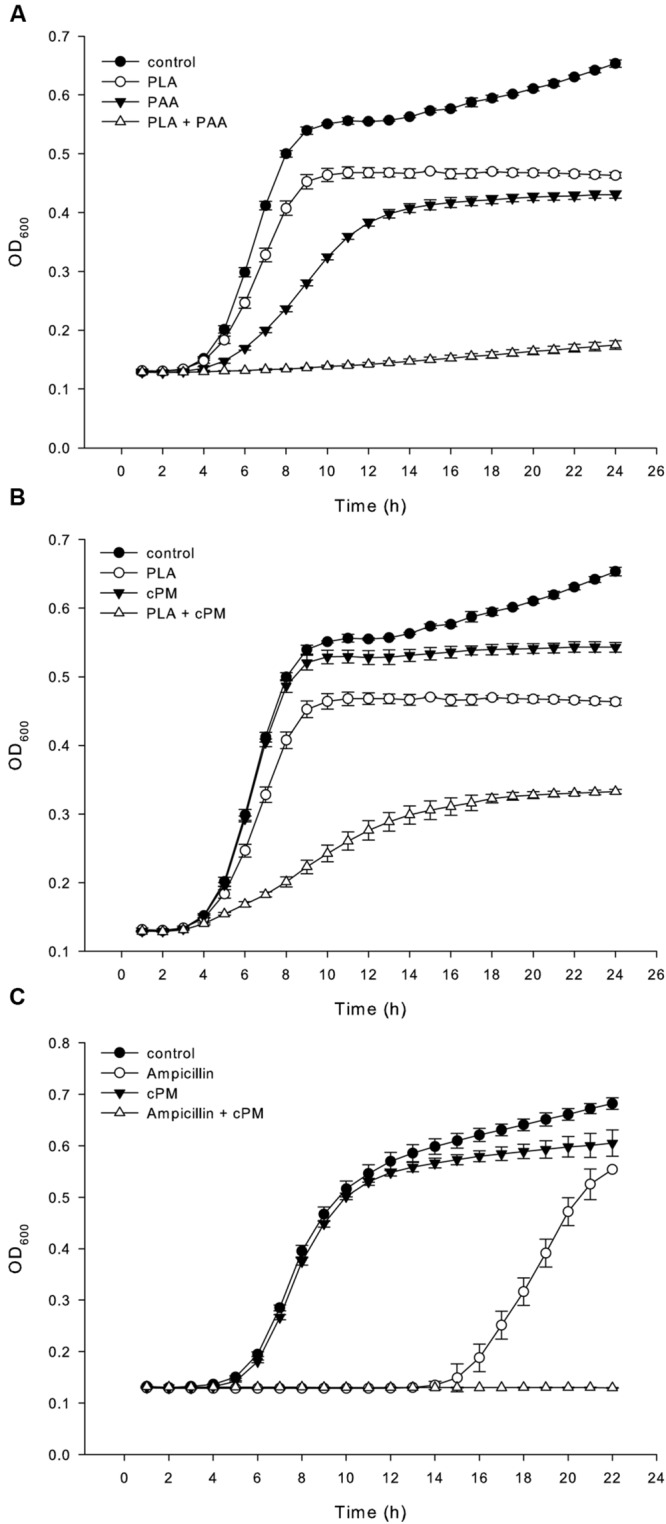
**Effect of combined compounds on antimicrobial activity.** Effect of PLA (0.5 g/l) and PAA (1 g/l) alone and combined **(A)**, and effect of the diketopiperazine cPM (1 g/l) and PLA (0.5 g/l) alone and combined **(B)** on the growth of *S. aureus*. **(C)** Effect of ampicillin (10 mg/l) and cPM (100 mg/l) alone and combined on the growth of *S. aureus* MRSA strain B335466. Data are presented as mean values ± standard deviations of three biological replicates performed in triplicate.

The effect of combinations of the identified compounds (concentrations from 0.002 to 2 g/l) on the antimicrobial activity was determined against *S. aureus, P. aeruginosa* and *C. albicans* (Supplementary Table S3). The combination of PAA and PLA decreased the MIC value to 1 g/l against *S. aureus* (**Figure 5A**). The antimicrobial activity of PLA was additionally increased in the presence of the diketopiperazine cPM (**Figure 5B**). All antimicrobial active compounds except cPM and cFP showed increased antimicrobial activity against *S. aureus* and *P. aeruginosa* if combined with PAA. Combination of cPM, PAA and PLA lead to synergistic increase of antimicrobial activity of PLA (Supplementary Table S3).

Furthermore we studied the ability of PAA, cPM and cFP to increase antimicrobial activity of ampicillin against both ESBL and MRSA strains. The combination of cPM (100 mg/l) with ampicillin decreased the MIC value from 100 to 10 mg/l against the MRSA strain B335466 (**Figure 5C**). The combination of PAA (1 g/l) and ampicillin showed a weak synergistic effect on the MRSA strain B335466 decreasing the MIC from 100 to 50 mg/l. Additionally the MRSA strain B337919 was less susceptible to the synergistic activity of cPM (2 g/l) and ampicillin which resulted in a decrease of MIC value from 100 to 50 mg/l. No synergistic or additive effect was observed against the ESBL *E. coli* strain and the ESBL *K. pneumoniae* strain (Supplementary Table S3).

## Discussion

Recent studies have shown that small molecule effectors like VPA are able to induce “cryptic” secondary metabolite production in fungi (Shwab et al., 2007; Cole, 2008; Cichewicz, 2010). The ability of VPA to induce antimicrobial activity in a broad range of fungi and to trigger the expression of secondary metabolite related polyketide synthases has been shown by our group before (Zutz et al., 2013, 2014). Thus, the goal of this study was to identify the “cryptic” antimicrobial compounds underlying the activity observed in the VPA treated fungal culture of *D. microsporus*. However, if the induction of antimicrobial activity is linked to the ability of VPA to modulate chromatin structure or other effects remains to be clarified. To better describe the influence of VPA on the production of “cryptic” antimicrobial compounds, VPA treated fungal culture were used for bioactivity driven fractionation.

Seven “cryptic” antimicrobial active compounds could be isolated (PAA, 4FP, IAA, PLA, cPM, ICA and cFP). Interestingly all isolated compounds except the two diketopiperazines are associated with the primary metabolism of fungi. PAA, ICA and IAA are phytohormones (Gustafson, 1941; Anaya et al., 1992). This indicates that the habitat of the fungus may not only be linked to decaying plant material and feces, but that this fungus could have the potential to infest plants. During the growth of plants a complex pattern of auxin-related compounds like IAA and ICA regulates physiological and developmental processes. There are suggestions that PAA is linked also to the auxin biosynthesis pathway. In *Azospirillum brasilense* it has been shown that the PAA synthesis is mediated by the indole-3-pyruvate decarboxylase (IpdC), a key enzyme of IAA production (Somers et al., 2005). PAA furthermore increased like IAA and other auxins the expression of the *ipdC* gene (Somers et al., 2005).

There is evidence that the cohabitation of fungi and bacteria (either endophytic or symbiotic) influence the production of these compounds in the plant. This can also lead to tumor or gall formation (Ditengou and Lapeyrie, 2000; Vasanthakumar and McManus, 2004; Reineke et al., 2008; Xin et al., 2009; Zuniga et al., 2013; Duca et al., 2014). ICA has been shown to be part of the priming response of higher plants to the infestation of *Plectosphaerella cucumerina* (Gamir et al., 2012). In the plant pathogenic/saprophytic fungus *Rhizoctonia solani* the PAA production has been identified as major component for the virulence of the fungus (Mandava et al., 1980). Trp is known to be one of the main substrates for the IAA biosynthesis pathway in plants (Magnus et al., 1982). Thus we investigated the influence of Trp alone and in combination with VPA on the production of the indole related compounds identified in this study. Trp doubles the production of IAA and ICA. These data suggest that a functional indole biosynthesis pathway may be present in the fungus.

Another strong indication for the potential ability of *D. microsporus* to infest plants is the production of 4FP, which was slightly increased if the fungus was treated with VPA. 4FP is known as phytotoxic metabolite of fungi and has been isolated from *Discula quercina* and *Ceratocystis* spp. Additionally 4FP has been identified together with PAA as a major phytotoxic compound produced by the fungus *Monilia* sp. (Ayer et al., 1986; Maddau et al., 2011).

Phenyllactic acid, originally isolated from bacteria, has so far only been detected in one fungus, *Geotrichum candidum*, which is used as biocontrol agent in cheese ripening (Dieuleveux et al., 1998). *Geotrichum candidum* is also able to produce IAA. In this study PLA is the only compound which was not detectable in the untreated fungal culture. Thus VPA increased the production of an otherwise silent antimicrobial “cryptic” compound. In a recent study the influence of aromatic amino acids including Trp on the production of PLA and IAA in *Geotrichum candidum* was investigated (Naz et al., 2013). It has been shown that Trp addition increased the production of PLA and IAA. However, in *D. microsporus* Trp showed no measurable effect on PLA production. The observation that Trp does not increase production of PLA but of IAA considerably indicates that either PLA is linked to a different biosynthesis pathway in *D. microsporus* or that the amount of produced PLA is below the limit of detection.

The majority of isolated compounds derived from the primary metabolism. These compounds have been secreted (or leaked) into the media. Secretion of metabolites is linked to many biological functions like influencing the auxin levels of the host and disrupting of quorum sensing signals. Secreted metabolites are known to be main players in the complex growth interactions of microorganisms. The variety of discovered bioactive metabolites indicates a potentially larger number of secreted metabolites yet to be discovered.

Seven “cryptic” antimicrobial compounds were isolated in the mg/l range. Although the MIC values of the respective compounds ranged from 0.4 to 5 g/l we observed a strong antimicrobial activity of the VPA treated fungal culture. This indicates that the cocktail of all seven compounds was responsible for the observed antimicrobial activity.

The most abundant compound was PAA which was produced in almost 10-fold higher concentration under VPA treatment. PAA is produced by fungi and bacteria (Mayrand, 1979; Mayrand and Bourgeau, 1982; Ait Kettout and Rahmania, 2010) and has been linked to antifungal and antibacterial activity against a broad range of microorganisms including *S. aureus, K. pneumoniae, P. aeruginosa*, and *L. monocytogenes* (Hwang et al., 2001; Kim et al., 2004; Slininger et al., 2004). In *P. aeruginosa* it has also been shown to inhibit quorum sensing (Musthafa et al., 2012).

Combination of ICA and IAA displayed increased activity against *S. aureus* but not against *P. aeruginosa*. PAA lead to an increase of antimicrobial activity of IAA, ICA, PLA and 4FP against *S. aureus*. This suggests that PAA could be able to potentiate antimicrobial activity. However, combinations of the indole related compounds, at the respective concentrations discovered in the fungus, did not result in the observed antimicrobial activity of the extracted fungal supernatants.

Additionally two diketopiperazines cFP and cPM were isolated. Diketopiperazines are cyclic dipeptides described in bacteria, which are mainly synthesized through non-ribosomal peptide synthetases activity and to lesser extend through cyclodipeptide synthases activity (Gu et al., 2013). Thus their synthesis is linked to the secondary metabolism. cPM was first and so far only isolated from an *P. aeruginosa* strain associated with the antarctic sponge *Isodictya setifera* (Mottram and Taylor, 2010; Neil et al., 2010). Previous characterizations of this compound were associated with processed food. In this study we demonstrated the first natural isolation of this rare compound from a fungus. The cyclic dipeptide family contains several bioactive compounds and they have been studied to determine their antimicrobial activity (Kumar et al., 2014). Proline based dipeptides are among the most potent cyclic dipeptides. The production of both diketopiperazines was induced by VPA treatment up to five fold. cFP, which is produced by bacteria and fungi, is involved in quorum sensing in gram negative bacteria (Holden et al., 2000). In the studies describing the first isolation of cPM and cFP no antimicrobial activity of either against *Bacillus subtilis, S. aureus* and *Micrococcus luteus* was reported (Jayatilake et al., 1996; Thomas et al., 2010). However, antimicrobial activity of cFP against higher fungi (MIC of 20 g/l) has been described (Strom et al., 2002). In this study we could show that cPM is antimicrobial active due to its growth inhibitory activity (2 g/l) against the tested microorganisms.

The combination of the isolated antimicrobial compounds with cPM and to lesser extent with cFP resulted in increased antimicrobial activity of 4FP, IAA and PLA. Furthermore we could show that cPM and to lesser extend PAA are able to enhance ampicillin activity against two resistant MRSA strains decreasing the MIC value to 10 mg/l. The ability of cPM to increase antimicrobial activity of iminpenem has recently been shown against a range of non-resistant medically important pathogens (Kumar et al., 2014).

We concluded that the observed antimicrobial activity of the VPA treated fungal culture against *S. aureus* is likely the result of PAA, cPM and cFP potentiating the activity of IAA, ICA, PLA and 4FP together with the additional effect of all compounds.

## Conclusion

We could show that VPA treatment is a potent tool for induction of “cryptic” antimicrobial compound production in fungi and that the induced compounds are not exclusively linked to the secondary metabolism. Furthermore this is the first discovery of the rare diketopiperazine cPM in fungi. Additionally we could show that cPM and PAA have the ability to potentiate antimicrobial activity of ampicillin against ampicillin resistant pathogens. This ability combined with low cytotoxicity makes cPM and to lesser extend PAA ideal candidates for combination therapies against resistant pathogens.

## Author Contributions

CZ, BK, KR, RS, MW, and JS contributed to the design of the work; CZ, MB, AP, BK, and AG-M were involved in the acquisition of the data; CZ, AP, MB, KR, JS, MW, RS, BK, AGM contributed to the analysis and interpretation of the data and CZ, KR, MW, and JS were involved in writing the manuscript and all authors revised the manuscript and approved the final version of the manuscript.

## Conflict of Interest Statement

The authors declare that the research was conducted in the absence of any commercial or financial relationships that could be construed as a potential conflict of interest.
