# Supplementary material for: Valproic Acid Induces Antimicrobial Compound Production in Doratomyces microspores
Source: Front Microbiol. 2016 Apr 13;7:510. doi: 10.3389/fmicb.2016.00510 (PMC4829596; doi:10.3389/fmicb.2016.00510)
Supplement: Supplementary file 1 [file Data_Sheet_1.PDF]

*Supplementary Material*

# Valproic acid induces antimicrobial compound production in *Doratomyces microsporus*

Christoph Zutz[1,2], Markus Bacher[6], Alexandra Parich[5], Bernhard Kluger[2,5], Agnieszka Gacek-Matthews[3], Rainer Schuhmacher[5], Martin Wagner[1], Kathrin Rychli[1*], Joseph Strauss[2,3,4]

*** Corresponding author:** Kathrin Rychli, Kathrin.rychli@vetmeduni.ac.at

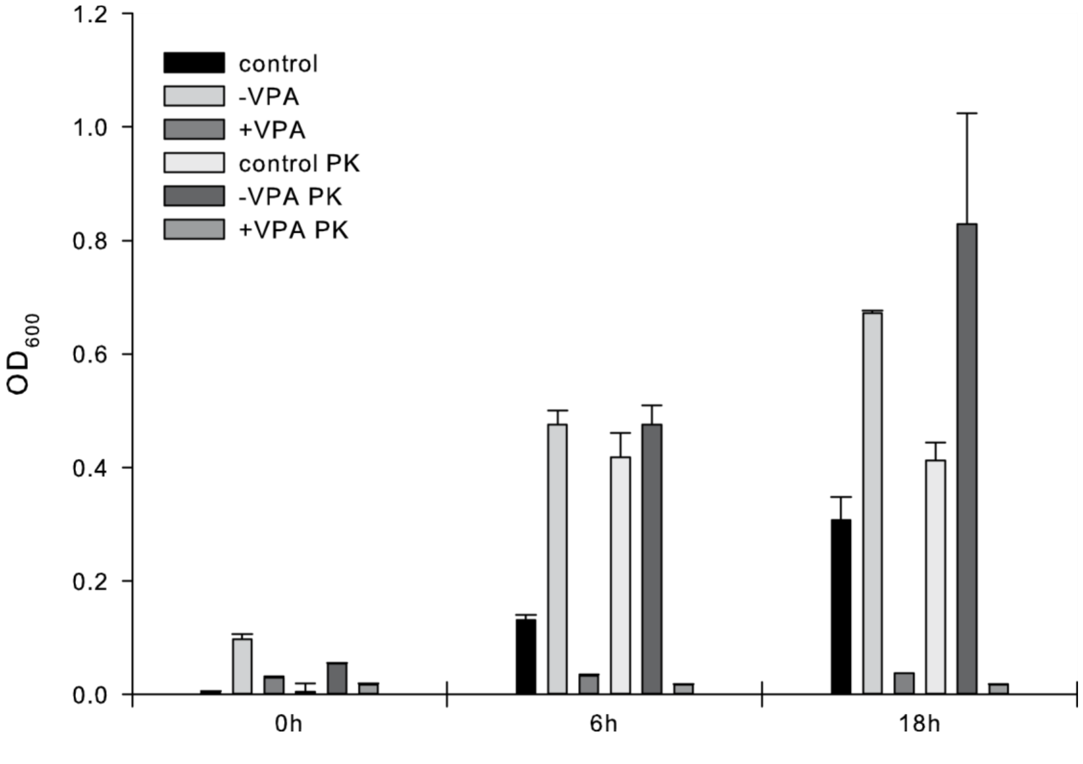


**Figure S1** Influence of Proteinase K digestion on the antimicrobial activity of the fungal extract. Fungal extract (-VPA) and fungal extract treated with VPA (+VPA) was incubated with 1 g/l proteinase K (-VPA PK; +VPA PK) for 24h at 37°C. Controls include *S. aureus* cells grown without fungal extract (control) and *S. aureus* cells grown in the presence of 1 g/l proteinase K (control PK). Data is presented as mean values ± standard deviations of three biological replicates performed in triplicate.

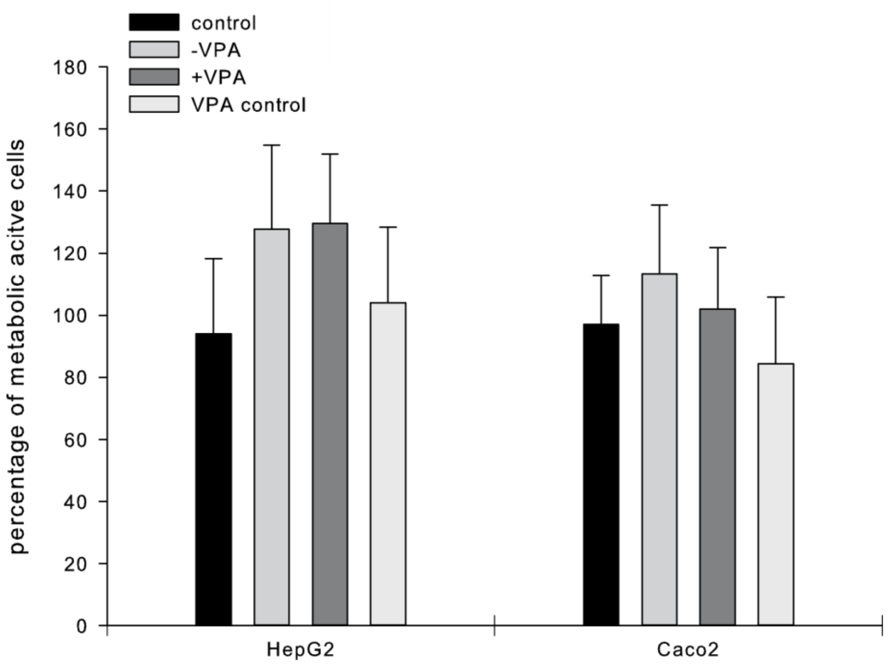


**Figure S2** Percentage of metabolic active cells human intestinal epithelial Caco2 and hepatocytic HepG2 measured incubated with the VPA treated (+VPA) and untreated extract (-VPA) of *D. microsporus* of cells. Controls include cells incubated with media (control) and media containing VPA (VPA control) for 24 h. Data are presented as mean values ± standard deviations of three biological replicates performed in triplicate.

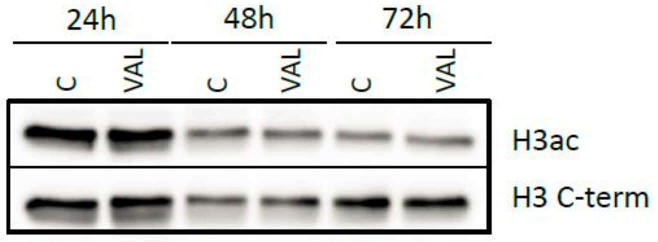


**Figure S3** Histone 3 acetylation (H3ac) levels of untreated (C) and VPA treated (VAL) *D. microsporus* after 24, 48 and 72 hours of incubation.

**Table S1: NMR shifts of isolated compounds**

N-(5 hydroxypentyl)acetamide

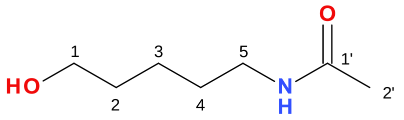


| MeOD | $^{1}$H | $^{13}$C |
|---|---|---|
| 1 | 3.55, t, 2H, *J* = 6.5 Hz | 62.76 |
| 2 | 1.55, m, 2H | 33.24 |
| 3 | 1.39, m, 2H | 24.26 |
| 4 | 1.52, m, 2H | 30.14 |
| 5 | 3.16, t, 2H, *J* = 7.0 Hz | 40.45 |
| 1´ | - | 173.18 |
| 2´ | 1.92, s, 3H | 22.51 |

5-acetamido pentanoic acid

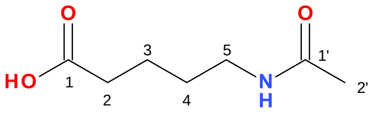


| MeOD | $^{1}$H | $^{13}$C |
|---|---|---|
| 1 | - | 177.29 |
| 2 | 2.32, t, 2H, *J* = 7.2 Hz | 34.44 |
| 3 | 1.62, m, 2H | 23.34 |
| 4 | 1.53, m, 2H | 29.78 |
| 5 | 3.17, t, 2H, *J* = 6.8 Hz | 40.06 |
| 1´ | - | 173.24 |
| 2´ | 1.93, s, 3H | 22.51 |

N-(5 hydroxypentyl)acetamide

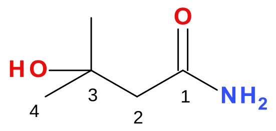


| MeOD | $^{1}$H | $^{13}$C |
|---|---|---|
| 1 | - | 175.32 |
| 2 | 2.46, s, 2H | 48.15 |
| 3 | - | 70.11 |
| 4 | 1.30, s, 6H | 29.31 |

p-hydroxylbenzaldehyd

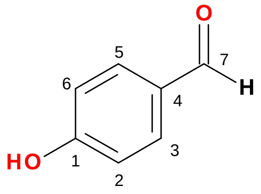


| MeOD | $^{1}$H | $^{13}$C |
|---|---|---|
| 1 | - | 165.12 |
| 2, 6 | 6.91, d, 2H, $J$ = 8.7 Hz | 116.85 |
| 3, 5 | 7.76, d, 2H, $J$ = 8.7 Hz | 133.42 |
| 4 | - | 130.26 |
| 7 | 9.75, s, 1H | 192.84 |

| Phenylacetic acid (PAA) | | |
|---|---|---|
| 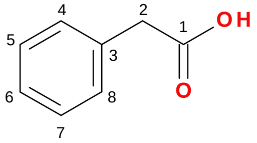 | | |
| **MeOD** | $^{1}$**H** | $^{13}$**C** |
| 1 | - | 175.68 |
| 2 | 3.59 (s, 2H) | 42.03 |
| 3 | - | 136.14 |
| 4, 8 | 7.27 (m, 2H) | 130.34 |
| 5, 7 | 7.30 (m, 2H) | 129.44 |
| 6 | 7.23 (m, 1H) | 127.87 |

| Phenyllactic acid (PLA) | | |
|---|---|---|
| 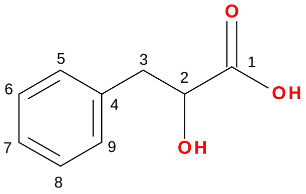 | | |
| **MeOD** | $^{1}$**H** | $^{13}$**C** |
| 1 | - | 177.43 |
| 2 | 4.31 (dd, 1H, *J* = 8.0 + 4.2 Hz) | 72.98 |
| 3 | 3.10 (dd, 1H, *J* = 13.9 + 4.2 Hz) | 41.68 |
| 4 | - | 139.03 |
| 5, 9 | 7.20 - 7.30 (m) | 130.58 |
| 6, 8 | 7.20 - 7.30 (m) | 129.21 |
| 7 | 7.19 (m, 1H) | 127.46 |

Indole-3-acetic acid (IAA)

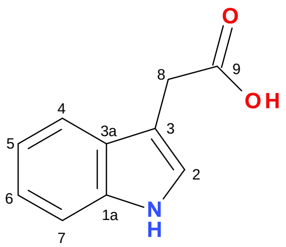


| MeOD | $^{1}$H | $^{13}$C |
|---|---|---|
| 1a | - | 138.02 |
| 2 | 7.16 (s, 1H) | 124.59 |
| 3 | - | 109.04 |
| 3a | - | 128.70 |
| 4 | 7.54 (br.d, 1H, *J* = 7.8 Hz) | 119.44 |
| 5 | 7.01 (ddd, 1H, *J* = 7.8 + 7.2 + 1.0 Hz) | 119.78 |
| 6 | 7.09 (ddd, 1H, *J* = 8.0 + 7.2 + 1.0 Hz) | 122.40 |
| 7 | 7.34 (br.d, 1H, *J* = 8.0 Hz) | 112.20 |
| 8 | 3.72 (s, 2H) | 32.11 |
| 9 | - | 176.64 |

Indol-3-carboxylic acid (ICA)

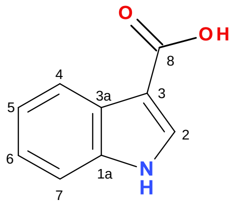


| MeOD | $^{1}$H | $^{13}$C |
|---|---|---|
| 1a | - | 137.9 |
| 2 | 7.94 (s, 1H) | 133.1 |
| 3 | - | 108.6 |
| 3a | - | 127.4 |

| | | |
|---|---|---|
| 4 | 8.07 (br.d, 1H, *J* = 6.8 Hz) | 121.8 |
| 5 | 7.16 (m, 1H) | 122.1 |
| 6 | 7.20 (m, 1H) | 123.3 |
| 7 | 7.43 (br.d, 1H, *J* = 7.0 Hz) | 112.6 |
| 8 | - | 169.2 |

Cyclo-(pro-met) cPM

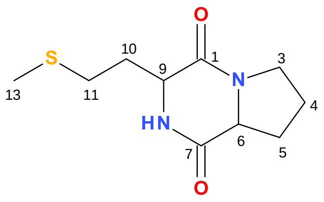


| **CDCl3** | **$^{1}$H** | **$^{13}$C** |
|---|---|---|
| 1 | - | 165.34 |
| 3 | 3.61 + 3.55 (each 1H, m) | 45.49 |
| 4 | 2.02 + 1.90 (each 1H, m) | 22.66 |
| 5 | 2.36 + 2.12 (each 1H, m) | 28.20 |
| 6 | 4.11 (br.t, 1H, *J* = 8.0 Hz) | 59.01 |
| 7 | - | 170.29 |
| 9 | 4.21 (br.t, 1H, *J* = 5.7 Hz) | 54.68 |
| 10 | 2.41 + 2.00 (each 1H, m) | 28.85 |
| 11 | 2.69 (br.t, 1H, *J* = 6.0 Hz) | 30.31 |
| 13 | 2.13 (s, 3H) | 15.30 |
| NH | 6.68 (br.s, 1H) | - |

| **MeOD** | **$^{1}$H** | **$^{13}$C** |
|---|---|---|
| 1 | - | 167.89 |
| 3 | 3.55 + 3.49 (each 1H, m) | 46.42 |
| 4 | 2.01 + 1.93 (each 1H, m) | 23.08 |
| 5 | 2.31 + 2.00 (each 1H, m) | 29.28 |
| 6 | 4.24 (m, 1H) | 60.31 |

| | | |
|---|---|---|
| 7 | - | 172.56 |
| 9 | 4.29 (br.t, 1H, *J* = 5.7 Hz) | 55.35 |
| 10 | 2.21 + 2.08 (each 1H, m) | 30.24 |
| 11 | 2.62 (m, 2H) | 30.37 |
| 13 | 2.10 (s, 3H) | 15.10 |

Cyclo (-phe-pro) cFP

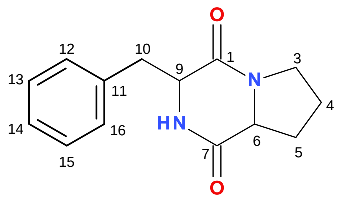


| **MeOD** | $^{1}$**H** | $^{13}$**C** |
|---|---|---|
| 1 | - | 166.91 |
| 3 | 3.54 + 3.37 (each 1H, m) | 45.96 |
| 4 | 1.80 (2H, m) | 22.70 |
| 5 | 2.10 + 1.25 (each 1H, m) | 29.38 |
| 6 | 4.07 (ddd, 1H, *J* = 10.7 + 6.4 + 1.9 | 60.08 |
| 7 | - | 170.92 |
| 9 | 4.44 (dt, 1H, *J* = 5.0 + 1.9 Hz) | 57.69 |
| 10 | 3.17 (d, 2H, *J* = 5.0 Hz) | 38.21 |
| 11 | - | 137.35 |
| 12, 16 | 7.15 – 7.35 (m) | 131.04 |
| 13, 15 | 7.15 – 7.35 (m) | 129.45 |
| 14 | 7.15 – 7.35 (m) | 128.07 |

**Table S2**

| | Compound [mg/ml] | | | | | | |
|---|---|---|---|---|---|---|---|
| | **PAA [2mg/ml]** | **4FP [2mg/ml]** | **ICA [2mg/ml]** | **IAA [0.2mg/ml]** | **PLA [2mg/ml]** | **cPM [2mg/ml]** | **cFP [2mg/ml]** |
| **% of living Caco2 cells** | 6.53 ± 0.54 | 25.94 ± 4.45 | 8.36 ± 3.40 | 8.61 ± 2.47 | 19.65 ± 5.75 | 4.77 ± 3.72 | 4.28 ± 2.3 |
| **% of metabolic active Caco2 cells** | 113.54 ± 19.78 | 32.06 ± 29.24 | 97.59 ± 14.28 | 103.64 ± 13.8 | 41.48 ± 23.56 | 99.18 ± 11.31 | 106.66 ± 9.52 |
| **% of living HepG2 cells** | 6.99 ± 4.45 | 7.86 ± 4.82 | 4.86 ± 1.77 | 7.27 ± 1.77 | 54.63 ± 18.02 | 3.99 ± 1.80 | 5.11 ± 1.44 |
| **% of metabolic active HepG2 cells** | 122.9 ± 12.06 | 57.33 ± 4.4.47 | 122.3 ± 26.94 | 108.28 ± 17.98 | 66.01 ± 9.39 | 107.47 ± 11.64 | 116.63 ± 24.03 |

**Table S3.** Effect of the combinations of the isolated compounds on the antimicrobial activity against *S. aureus, P. aeruginosa* and *C. albicans*

***S.aureus***

| | **PAA** | **4FP** | **ICA** | **IAA** | **PLA** | **cPM** | **cFP** |
|---|---|---|---|---|---|---|---|
| **PAA** | | ++ | ++ | ++ | ++ | - | - |
| **4FP** | ++ | | - | - | - | ++ | ++ |
| **ICA** | ++ | - | | - | - | - | - |
| **IAA** | ++ | - | - | | + | ++ | - |
| **PLA** | ++ | - | - | + | | ++ | - |
| **cPM** | - | ++ | - | ++ | ++ | | - |
| **cFP** | - | ++ | - | - | - | - | |

***P. aeruginosa***

| | **PAA** | **4FP** | **ICA** | **IAA** | **PLA** | **cPM** | **cFP** |
|---|---|---|---|---|---|---|---|
| **PAA** | | ++ | ++ | ++ | ++ | - | + |
| **4FP** | ++ | | - | - | - | ++ | + |
| **ICA** | ++ | - | | + | - | ++ | + |
| **IAA** | ++ | - | + | | + | - | - |
| **PLA** | ++ | - | - | + | | - | - |
| **cPM** | - | ++ | ++ | - | - | | - |
| **cFP** | + | + | + | - | - | - | |

***C. albicans***

| | **PAA** | **4FP** | **ICA** | **IAA** | **PLA** | **cPM** | **cFP** |
|---|---|---|---|---|---|---|---|
| **PAA** | | ++ | - | ++ | - | ++ | ++ |
| **4FP** | ++ | | - | - | - | - | - |
| **ICA** | - | - | | - | - | ++ | ++ |
| **IAA** | ++ | - | - | | - | - | - |
| **PLA** | - | - | - | - | | - | ++ |
| **cPM** | ++ | - | ++ | - | - | | - |
| **cFP** | ++ | - | ++ | - | ++ | - | |

++synergistic effect, + additive effect, - no effect,
